# Altered anisotropy and diffusivity of medulla oblongata and spinal cord in adolescent idiopathic scoliosis

**DOI:** 10.1186/1748-7161-10-S1-O45

**Published:** 2015-01-19

**Authors:** Winnie CW Chu, Youyong Kong, Lin Shi, Steve Hui, Defeng Wang, Min Deng, Yong Qiu, Jack Cheng

**Affiliations:** 1 Department of Imaging and Interventional Radiology, The Chinese University of Hong Kong, Hong Kong; 2 Department of Medicine and Therapeutics, The Chinese University of Hong Kong, Hong Kong; 3 Spine Surgery, The Affiliated Drum Tower Hospital of Nanjing University Medical School, Nanjing, China; 4 Department of Orthopaedics & Traumatology, Chinese University of Hong Kong, Hong Kong; 5 Joint Scoliosis Research Center of the Chinese University of Hong Kong and Nanjing University, China

## Objective

Abnormal somatosensory evoked potentials (SEP) have been demonstrated above the C5-6 level of the spinal cord. Together with observation of tonsillar ectopia and relatively tethered cord, we hypothesize that there is abnormal changes of the white matter integrity along the brainstem and spinal cord. The objective of this study is to utilize the advanced diffusion tensor imaging (DTI) to examine the potential white matter changes of medulla oblongata and spinal cord.

## Materials and methods

Thirteen AIS girls with right thoracic curves and thirteen age-matched healthy girls were recruited. DTI of both the brain and the whole spinal cord were acquired on a 3T MRI scanner. Region of interests were manually defined for medulla oblongata and along each intervertebral segment of the spinal cord. Mean values of fractional anisotropy (FA) and mean diffusivity (MD) were computed at the defined regions. Between-group comparisons were performed using the one way of analysis of variance.

## Results

Significant decreased FA values and increased MD values were found at the medulla oblongata, C1-2, C2-3, C3-4 and C4-5 segments of the spinal cord in AIS patients compared to normal subjects. No significant difference was found in other segments. There was significant correlation found between tonsillar level and FA value at C4/5 level in AIS patients only.

## Conclusion

The DTI findings support our proposed hypothesis of disturbed white matter integrity within the brainstem and spinal cord, which together with low-lying cerebellar tonsils are associated features of tethered cord in AIS. The changes in FA and MD are in line with abnormal SEP observed clinically.

